# The “Undetermined Significance” of ^18^F-FDG PET/CT or PET/MRI in Patients with Monoclonal Gammopathy of Undetermined Significance (MGUS)

**DOI:** 10.3390/medicina57080856

**Published:** 2021-08-23

**Authors:** Giorgio Treglia, Francesco Bertagna, Domenico Albano

**Affiliations:** 1 Clinic of Nuclear Medicine, Imaging Institute of Southern Switzerland, Ente Ospedaliero Cantonale, 6500 Bellinzona, Switzerland; 2 Department of Nuclear Medicine and Molecular Imaging, Lausanne University Hospital, 1011 Lausanne, Switzerland; 3 Academic Education, Research and Innovation Area, General Directorate, Ente Ospedaliero Cantonale, 6500 Bellinzona, Switzerland; 4 Faculty of Biology and Medicine, University of Lausanne, 1011 Lausanne, Switzerland; 5 Faculty of Biomedical Sciences, Università della Svizzera italiana, 6900 Lugano, Switzerland; 6 Department of Nuclear Medicine, University of Brescia and Spedali Civili Brescia, 25123 Brescia, Italy; francesco.bertagna@unibs.it (F.B.); domenico.albano@unibs.it (D.A.)

**Keywords:** PET, FDG, MGUS, myeloma, monoclonal gammopathy, plasma cell, evidence-based, recommendation, imaging

## Abstract

Monoclonal gammopathy of undetermined significance (MGUS) is a highly prevalent condition with the possible risk of progression to multiple myeloma (MM) or a lymphoproliferative neoplasm in a small percentage of patients. Fluorine-18 fluorodeoxyglucose (^18^F-FDG) positron emission tomography/computed tomography (PET/CT) or positron emission tomography/magnetic resonance imaging (PET/MRI) are imaging methods increasingly used in patients with MM. The aim of this communication is to underline that, taking into account current evidence-based data, compared to MM the role of ^18^F-FDG PET/CT or PET/MRI in MGUS is still undetermined and more studies should be performed before suggesting ^18^F-FDG PET/CT or PET/MRI for evaluation of MM progression in patients with MGUS.

## 1. Introduction

Monoclonal gammopathy of undetermined significance (MGUS) is an asymptomatic disorder. Diagnostic criteria for MGUS are the following: serum M-protein <30 g/L, bone marrow clonal plasma cells <10%, absence of end-organ damage related to plasma cell neoplasms and absence of diseases producing an M-protein. MGUS is considered a preneoplastic plasma cell disorder that does not always progress to overt malignancy [1,2,3,4]. This disorder affects about 3.5% of the population with an age >50 years; progression of MGUS to MM or other related neoplasms occurs at a rate of about 1% per year [1,2,3,4].

Multiple myeloma (MM) is a common haematological neoplasm and typically starts as asymptomatic precursor conditions, such as MGUS or smouldering multiple myeloma (SMM) [5,6].

Fluorine-18 fluorodeoxyglucose (^18^F-FDG) positron emission tomography/computed tomography (PET/CT) and positron emission tomography/magnetic resonance imaging (PET/MRI) are hybrid imaging methods that allow the evaluation of elevated glucose metabolism in malignancies. In the last year there has been increasing interest in ^18^F-FDG PET/CT or PET/MRI for plasma cell disorders and multiple applications of these imaging methods were suggested including distinguishing SMM from active MM, confirmation of solitary plasmacytoma, staging, monitoring and assessing treatment response and detection of residual disease following treatment in MM [7,8,9]. Furthermore, ^18^F-FDG PET/CT or PET/MRI are reliable techniques for assessing bone marrow and skeletal involvement and for predicting outcomes of MM. Conversely, the role of ^18^F-FDG PET/CT or PET/MRI in patients with MGUS seems controversial [10].

In this communication we would like to briefly discuss current international consensus recommendations and evidence-based data about the role of ^18^F-FDG PET/CT or PET/MRI in patients with MGUS.

## 2. Consensus Recommendations

The 2019 International Myeloma Working Group (IMWG) consensus document on imaging in monoclonal plasma cell disorders recommends whole-body CT (or conventional skeletal survey or whole-body MRI as alternatives if whole-body CT is not available) to rule out MM in suspected high-risk MGUS. In MGUS patients in whom there is a concern for MM development, with equivocal findings on a whole-body CT or conventional skeletal survey, the IMWG recommends whole-body MRI or MRI of the spine and pelvis if whole-body MRI is not available. About ^18^F-FDG PET/CT, this method is recommended if whole-body CT is positive in patients with MGUS [11]. In other words, whole-body CT is the preferred imaging method in patients with high-risk MGUS, but ^18^F-FDG PET/CT or PET/MRI could be used as the next-step imaging method for further investigation of equivocal findings according to this consensus recommendation.

## 3. Literature Data

Surprisingly, despite the recommendations cited above, literature data about the possible role of ^18^F-FDG PET/CT or PET/MRI in patients with MGUS are scarce.

Only one recently published prospective study (including 390 MGUS patients) evaluated the benefit of ^18^F-FDG PET/CT in the early detection of various accompanying disorders in MGUS patients [12]. On ^18^F-FDG PET/CT scans, the presence of focal or diffuse areas of abnormal increased radiopharmaceutical uptake was described in about 10% of MGUS patients. The most frequent abnormal findings were lymphadenopathies (3.8%), thyroid diseases (2.1%), rheumatic diseases (1.8%), and other solid malignancies (1.5%). Based on these findings and since MGUS may be connected to an increased risk of developing haematological as well as solid malignancies or other disorders, the authors suggested the use of ^18^F-FDG PET/CT in all newly diagnosed MGUS patients. Theoretically, this diagnostic approach would allow for early detection of other serious diseases and prediction of the progression of MGUS to MM [12].

## 4. “Real-World” Scenario and Future Perspectives

We strongly believe that recommendations of new applications for ^18^F-FDG PET/CT or PET/MRI should follow the principles of evidence-based medicine and health technology assessment (HTA) [13,14]. In this regard, even in the presence of initial encouraging results [12] and expert recommendations [11], the role of ^18^F-FDG PET/CT or PET/MRI in MGUS is still undetermined.

Furthermore, several domains should be evaluated before recommending the use of an imaging method including the following: availability, clinical value, safety, costs, organisational, social and legal aspects. PET/CT or PET/MRI are relatively expensive diagnostic procedures with a reduced availability compared to other imaging techniques and with a relatively higher radiation exposure than many conventional diagnostic imaging examinations. The high prevalence of MGUS in the general population and the low rate of progression of MGUS to serious malignancies should be taken into account before suggesting the use of ^18^F-FDG PET/CT or PET/MRI in all newly diagnosed MGUS patients. Overall, there are no data on the cost-effectiveness of a diagnostic approach including ^18^F-FDG PET/CT or PET/MRI in MGUS patients compared to a diagnostic approach without these methods, and more studies should be performed before suggesting ^18^F-FDG PET/CT or PET/MRI for the evaluation of MGUS patients and, in particular, for evaluating the progression of MGUS to MM.

## Data Availability

Data reported in this article are retrieved by PubMed (a free bibliographic database).

## References

[1] Lomas O.C., Mouhieddine T.H., Tahri S., Ghobrial I.M. (2020). Monoclonal Gammopathy of Undetermined Significance (MGUS)-Not So Asymptomatic after All. Cancers.

[2] Seth S., Zanwar S., Vu L., Kapoor P. (2020). Monoclonal Gammopathy of Undetermined Significance: Current Concepts and Future Prospects. Curr. Hematol. Malig. Rep..

[3] Ho M., Patel A., Goh C.Y., Moscvin M., Zhang L., Bianchi G. (2020). Changing paradigms in diagnosis and treatment of monoclonal gammopathy of undetermined significance (MGUS) and smoldering multiple myeloma (SMM). Leukemia.

[4] Rajkumar S.V., Dimopoulos M.A., Palumbo A., Blade J., Merlini G., Mateos M.V., Kumar S., Hillengass J., Kastritis E., Richardson P. (2014). International Myeloma Working Group updated criteria for the diagnosis of multiple myeloma. Lancet Oncol..

[5] van de Donk N.W.C.J., Pawlyn C., Yong K.L. (2021). Multiple myeloma. Lancet.

[6] Mann H., Katiyar V., Varga C., Comenzo R.L. (2021). Smoldering multiple myeloma-Past, present, and future. Blood Rev..

[7] Ulaner G.A., Landgren C.O. (2020). Current and potential applications of positron emission tomography for multiple myeloma and plasma cell disorders. Best Pract. Res. Clin. Haematol..

[8] Jamet B., Bailly C., Carlier T., Touzeau C., Nanni C., Zamagni E., Barré L., Michaud A.V., Chérel M., Moreau P. (2019). Interest of Pet Imaging in Multiple Myeloma. Front. Med..

[9] Vicentini J.R.T., Bredella M.A. (2021). Role of FDG PET in the staging of multiple myeloma. Skeletal Radiol..

[10] Jamet B., Bailly C., Carlier T., Touzeau C., Michaud A.V., Bourgeois M., Moreau P., Bodet-Milin C., Kraeber-Bodere F. (2020). Imaging of Monoclonal Gammapathy of Undetermined Significance and Smoldering Multiple Myeloma. Cancers.

[11] Hillengass J., Usmani S., Rajkumar S.V., Durie B.G.M., Mateos M.V., Lonial S., Joao C., Anderson K.C., García-Sanz R., Riva E. (2019). International myeloma working group consensus recommendations on imaging in monoclonal plasma cell disorders. Lancet Oncol..

[12] Sandecka V., Adam Z., Krejci M., Stork M., Rehak Z., Koukalova R., Sevcikova S., Brozova L., Kral Z., Mayer J. (2020). Diagnostic relevance of 18F-FDG PET/CT in newly diagnosed patients with monoclonal gammopathy of undetermined significance (MGUS): Single-center experience. Neoplasma.

[13] Fuchs S., Grössmann N., Ferch M., Wild C. (2019). Evidence-based indications for the planning of PET or PET/CT capacities are needed. Clin. Transl. Imaging.

[14] Treglia G., Sadeghi R. (2013). Meta-analyses and systematic reviews on PET and PET/CT in oncology: The state of the art. Clin. Transl. Imaging.

